# Modified Seed Growth of Iron Oxide Nanoparticles in Benzyl Alcohol — Optimization for Heating and Broad Stability in Biomedical Applications

**DOI:** 10.5772/60035

**Published:** 2014-01-01

**Authors:** Stanley E. Gilliland, Everett E. Carpenter, Michael D. Shultz

**Affiliations:** 1 Research and Development, Hunter Holmes McGuire VA Medical Center, Richmond, USA; 2 Department of Chemistry and Nanomaterials Core Characterization Facility, Virginia Commonwealth University, Richmond, USA

**Keywords:** biocompatible materials, cancer therapy, hyperthermia, benzyl alcohol, magnetic iron oxide nanoparticles

## Abstract

Iron oxide nanoparticles have received sustained interest for biomedical applications as synthetic approaches are continually developed for control of nanoparticle properties. However, many approaches focus solely on the material, rather than the complete optimization of synthesis and functionalization together to enhance translation into biological systems. Presented herein is a modified seed growth method designed for obtaining optimal nanoparticle properties and ease of surface functionalization for long term stability. With a one or two addition process, iron oxide nanoparticles were produced in crystallite sizes ranging from 5–15 nm using only benzyl alcohol and an iron precursor. In the functionalization process, concentration variations were required for stabilizing different nanoparticle sizes. Radio frequency induction heating experiments of various crystallite and hydrodynamic sizes verified that the heating efficiency greatly increased while approaching the 15 nm crystallite, and suggested an important role of the overall particle size on heating efficiency. Initial *in vitro* experiments with the functionalized nanoparticles showed success in providing hyperthermia-induced tumour cell killing without an increase in the temperature of the cell suspension medium. This demonstrates the potential for nanoparticle-based hyperthermia to provide a therapeutic effect while limiting normal tissue damage.

## 1. Introduction

In 1957 Gilchrist first reported the idea of using magnetic particles for hyperthermia treatment of tumours.[1] Hyperthermia is often divided into three temperature ranges that have various effects and interactions with other therapies.[2,3] Mild hyperthermia (39–42°C) is considered non-lethal temperature elevation and has been shown to sensitize tumours to chemotherapy or radiation by increased drug perfusion and oxygenation.[3-5] Moderate hyperthermia (41–46°C) causes cells to experience heat stress, promotes protein degradation and interrupts vital cellular processes eventually leading to apoptosis.[6-8] Thermoablation (>45°C) generates enough heat to directly destroy local tumour cells and tissues.[6,8,9] In order to produce these heating effects in tumours by magnetic particles, an external radiofrequency (RF) alternating current (AC) magnetic field is applied which heats magnetic particles by eddy currents, dielectric losses, or hysteretic heating.[1] The extent and rate of particle heating depends on the size, conductivity and magnetic properties of the material.[1,2,10-13] Gilchrist found that the frequency and field strength applied must be optimized to provide minimal heating of healthy tissue due to dielectric loss and maximize hysteretic heating of the magnetic particles.[1]

Research into magnetic particle-based hyperthermia has shifted from larger multi-domain particles, similar to Gilchrist's research, to smaller single-domain and superparamagnetic materials. The primary reason for this shift is that superparamagnetic nanoparticles are much more efficient at absorbing power to generate heat than microparticles.[2] Superparamagnetic nanoparticles generate heat by two mechanisms. The Néel relaxation mechanism generates heat through quickly altering the direction of magnetic moments with respect to the crystal lattice.[6] The Brownian mechanism generates heat as a result of the viscosity of the media resisting the physical rotation of the nanoparticles in an applied AC magnetic field.[14] The internal, Néel, and external, Brownian, sources of friction generate heat by loss of thermal energy.[14] Several factors can affect which mechanism of heating dominates, such as size, polydispersity, crystal structure, shape, and magnetic anisotropy. [14] However, it has been determined that the average crystallite size and narrow size distribution are two of the most important factors in maximizing energy absorption and heat production.[13,15] Other reasons for shifting to nanoparticles are that larger particles tend to be more invasive, have a higher potential for adverse damage to surrounding healthy cells, and do not generate uniform heating.[2] Furthermore, unlike larger magnetic particles, superparamagnetic nanoparticles do not retain their magnetism after removal of an external magnetic field and are thus less likely to aggregate, which prolongs blood circulation time.[16] Furthermore, with the combination of modern medicine and nanotechnology, nanoparticles can be specifically targeted to cancer cells to provide minimal invasiveness, and more local and confined heating.[2,6,17,18]

More specifically, iron oxide nanoparticles are a primary candidate for nanomedicine therapeutic applications in part due to their RF induction heating properties, as well as being biocompatible and biodegradable.[15,16] In addition, they can be classified as a theranostic agent[19-22] providing diagnostic imaging capabilities in the form of a magnetic resonance imaging (MRI) contrast[23,24] and therapeutic potential by means of magnetic fluid hyperthermia (MFH).[15,24,25] Superparamagnetic iron oxide nanoparticles functionalized with aminosilanes are currently undergoing clinical trials in Germany for MFH treatment of glioblastoma and prostate cancer.[17,25,26] The optimal iron oxide nanoparticles for heat generation by RF induction heating have been shown to have a crystallite size of 15–16 nanometres (nm).[27,28] This size gives the ideal combination of heating mechanisms with Néel relaxation being the dominant process.[29] Above this size, Brownian relaxation becomes the dominant heating mechanism, which yields lower heat generation.[29] Thus, optimization and investigation of iron oxide nanoparticle synthesis to control and obtain the best combination of crystallite size, particle size, monodispersity and magnetic properties is of continually growing interest.

There are currently several synthetic strategies to prepare superparamagnetic iron oxide nanoparticles using thermal decomposition methods.[30-33] The use of non-polar solvents allows for tunable size, high crystallinity, easy scale-up, and a narrow size distribution of nanoparticles, but they can be more difficult to phase transfer, functionalize and purify for biological applications. In addition, most of these approaches rely on several seed growth steps with intermediate wash steps, multiple solvents and capping agents to obtain the desired 15 nm crystallite size. [31,34] The synthesized nanoparticles then undergo rigorous phase transfer processes and surface functionalization methods to produce a biologically stable colloidal suspension. Magnetic fluid hyperthermia and nanomedicine in general rely heavily on maintaining the biological stability of the nanoparticles and the ability to carry targeting ligands to increase the affinity to tumour cells. [17,18,35] Thus, synthesizing nanoparticles that are easily functionalized, purified, stable in various media and can be further functionalized with targeting or therapeutic modalities is of paramount importance.

In this paper, we present a modified seed growth approach to produce nanoparticles with crystallite sizes of 5–15 nm and optimization of functionalization parameters for biological applications. The main purpose of the investigation is to optimize previous benzyl alcohol metal oxide synthesis[36-39] for a one-pot addition setup, with fewer washing steps, that produces iron oxide nanoparticles that are easier and more efficiently surface functionalized than previously reported methods.[31,34] Benzyl alcohol was used as the solvent, capping agent and reducing agent for the combined reduction and thermal decomposition of iron (III) acetylacetonate (Fe(acac)_3_). Benzyl alcohol is found naturally in oils of plants and used in cosmetic products, [40] as a flavour and fragrance additive, [40-42] and as a preservative of injectable drugs, [43] and has an overall low toxicity. Several synthetic parameters such as temperature, concentration, time and addition of extra iron precursors were investigated to optimize the iron oxide nanoparticles for magnetic fluid hyperthermia applications. The nanoparticle surface was functionalized with carboxymethylated polyvinyl alcohol (CMPVA), a biodegradable, cheap, and hydrophilic biopolymer.[35] This process was optimized to provide higher yield, stability in biologically relevant buffers and media, further ability for biofunctionalization, and allow for adjusting parameters to account for differences in nanoparticle size. Lastly, *in vitro* experiments with glioblastoma cells were conducted to highlight the potential of the resultant stable iron oxide nanoparticles for magnetic fluid hyperthermia.

## 2. Results and Discussion

### 2.1 Synthesis and Mechanistic Studies

**Figure 1.**
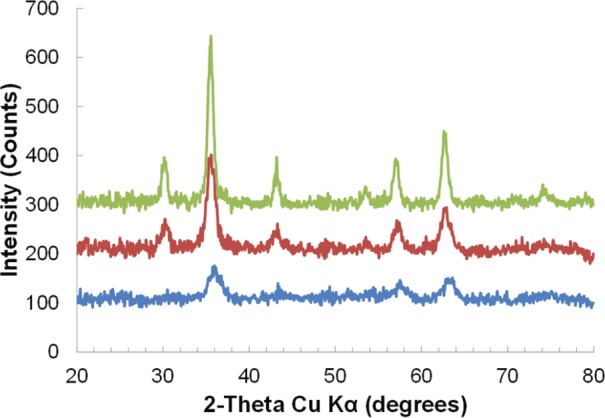
XRD analysis of reactions A2–24 under N2 (blue), A2–24 (red), and A2–24_B2–24 (green). XRD patterns are offset by 100 count increments.

Iron oxide nanoparticles were first synthesized in benzyl alcohol under nitrogen flow. The use of nitrogen or argon flow is often the standard method in the literature when carrying out thermal decomposition of Fe(acac)_3_ or iron carboxylate salts[24,34,44-49] This resulted in nanoparticles with a crystallite size of 5.43 ± 0.448 nm as calculated from the powder x-ray diffraction (XRD) pattern in Figure 1 using the Scherrer equation. The saturation magnetization (M_s_) was found to be 53.39 emu/g as measured by vibrating sample magnetometry (VSM) and mass corrected by thermal gravimetric analysis (TGA) data. As mentioned previously, the optimal crystallite size for magnetic nanoparticle-based induction heating has been shown to be around 15 nm. While this is not the overall particle size, and the exact relationship between particle size and heating is not clear, our goal was to use the benzyl alcohol-based synthesis to increase the crystallite and particle size into an optimal range for RF heating. Thus, our hypothesis was that the crystallite size could be increased by changing the reaction conditions from nitrogen flow to being open to air. Carrying out the reaction in the presence of air, A2–24, could facilitate the oxidation of benzyl alcohol to benzaldehyde and reduction of Fe(acac)_3_ at temperatures further below the start of thermal decomposition, similar to the mechanism of metal and metal oxide nanoparticle formation in glycols.[23,50-53] Starting the reaction at lower temperatures, where the temperature ramp rate is faster, would allow for fewer nuclei to form and a better separation of nucleation and growth phases; both of which would lead to larger nanoparticles and potentially a larger crystalline core.[15] This simple reaction parameter change resulted in iron oxide nanoparticles with a crystallite size of 8.33 ± 0.393 nm (Figure 1) and a M_s_ of 70.839 emu/g. Typically, the thermal decomposition of Fe(acac)_3_ starts to occur around 170–180°C depending on the solvent.[54] Nanoparticle formation, indicated by a colour change from dark red to black, initially occurred under nitrogen at 174.4°C after 31 minutes and the reaction solution appeared completely black after 40 minutes. In contrast, carrying out the reaction under air with identical heating rate and final temperature (Figure S1) resulted in an initial colour change at 169.4°C after 20 minutes and a completely black solution at 30 minutes. This indicates that the presence of oxygen leads to the reaction initiation occurring sooner in time and at a lower temperature, suggesting the possibility of an additional mechanism by which the iron oxide nanoparticles are forming in benzyl alcohol. In order to verify if the benzyl alcohol was acting as a reducing agent in this synthesis, FeCl_2_ was used as a precursor in place of the Fe(acac)_3_ with the addition of NaOH as in glycol synthetic methods.[23,51-53] This reaction produced magnetite under both air and N_2_ (Figure S2) confirming the presence of another mechanism of nanoparticle formation in benzyl alcohol other than thermal decomposition of Fe(acac)_3_. As with the Fe(acac)_3_ synthesis, the FeCl_2_ reaction under air had an initial colour change at 90.8°C compared to 99.3°C for N_2_, and turned completely black under air at 127.7°C versus 132.7°C for the reaction under N_2_ (Figure S1). Therefore, these results suggest that running the reaction under air promotes the earlier initiation of nucleation, giving further separation of nucleation and growth which led to the increase in crystallite size. From this mechanistic insight, all additional syntheses to increase size were carried out under air.

### 2.2 Modified Seed Growth and Synthetic Parameter Effects on Particle Size

With the goal of further increasing the crystallite size, monodispersity and particle size for optimal RF heating, a modified seed growth technique was investigated with different reaction times, temperatures, precursor concentrations, and multiple precursor additions. To keep track of the reaction conditions, the following naming system was utilized. A and B denote the first or second additions of Fe(acac)_3_ respectively and are separated by an underscore. The A and B are followed by numbers indicating the gram amount of Fe(acac)_3_ added at the respective addition. This is followed by a ‘-X’ with X indicating the time in hours the reaction proceeded before a subsequent addition. When temperature was investigated as a parameter, it is indicated by the number in parenthesis beside the reaction time. A2–24(175)_B2–24(175) for example indicates a reaction with 2 g of Fe(acac)_3_ initially added to 20 mL of benzyl alcohol in the presence of air. This was reacted for 24 hours at 175°C before a second addition of Fe(acac)_3_ which was then reacted for 24 hours at 175°C. If a temperature is not provided, all reactions were carried out using a heating mantle with identical power settings confirmed by a similar rate of reflux. These nanoparticles were characterized using XRD, dynamic light scattering (DLS), VSM, and TGA. The data are presented in Table 1.

**Table 1.** Saturation Magnetization, Heating Profile, and Average Size determined by VSM, Heating Induction, XRD, and DLS.

Reaction	Magnetization [emu/g]a)	RF Heating [(°C/min)/mg]b)	Crystallite Size [nm]	Hydrodynamic Diameter [nm]	PDIc)
A2–24	0.839	0.170	8.1	13.64	0.703
A2–24(175)	70.91	0.142	9.1	12.45	0.61
A2–24(195)	74.322	0.175	7.8	13.3	0.65
A4–24	68.99	0.134	8.3	14.68	0.2
A6–24	68.25	0.219	7.9	16.5	0.164
A2–24_B2–24	75.7	2.536	12.8	28.93	0.148
A2–24_B2[cool addition]-24	72.488	0.670	9.6	20.76	0.252
A2–24(175)_B2–24(175)	77.89	1.004	11.4	24.53	0.404
A2–24(185)_B2–24(185)	77.249	1.068	13.2	23.11	0.395
A2–24(195)_B2–24(195)	78.202	4.041	15.2	37.52	0.219
A2–2	60.6	0.032	6.3	10.93	0.311
A2–2_B2–2	62.85	0.069	5.9	17.88	0.447
A2–2_B2–24	76.1	0.211	9.2	20.07	0.373
A2–2_B4–2	62.8	0.102	8	15.43	0.258
A2–2_B4–24	72.18	0.212	9.4	17.72	0.304
A2–2_B6–24	75.56	0.639	11.1	19.42	0.368

a) Mass unit indicates grams of iron oxide nanoparticles corrected by TGA.

b) Mass unit indicates milligrams of Fe determined by Prussian blue assay.

c) Polydispersity Index (PDI) determined by DLS.

The first parameter investigated to increase the crystallite size was the initial Fe(acac)_3_ precursor concentration, as some synthetic methods in the literature use this parameter to increase the overall particle size.[55] Increasing the Fe(acac)_3_ amount by 2 g per reaction, (A2–24, A4–24 and A6–24) resulted in no significant change in crystallite size. This did however show an increase in the hydrodynamic diameter of 13.64 nm, 14.68 nm and 16.5 nm and polydispersity index (PDI) values of 0.703, 0.2 and 0.164 respectively (note: a lower PDI corresponds to a more monodisperse solution). The increase in overall particle size and decrease in PDI can be rationalized by the LaMer growth model.[56-58] Increasing the Fe precursor concentration leads to an increased rate of reaching the critical supersaturation concentration for nucleation and the critical limiting supersaturation level.[56,58] Upon reaching this critical supersaturation, a ‘burst’ nucleation event occurs, depleting the concentration of monomers for nucleation below the critical supersaturation limit and thus halting further nucleation.[56,58] Then, the reaction switches over to growth, with the remaining monomers in solution then growing on the nuclei by diffusion.[56,58] This provides a better separation of the nucleation and growth phases to increase the monodispersity and more available material for the growth phase leading to larger nanoparticles as seen in the results in Table 1.

The next parameter that was altered was reaction time, to determine its effect on the resultant particle characteristics. A time of 2 hours was chosen to give a significantly shorter reaction as compared to 24 hours, while ensuring that the reaction had ample time to turn completely black, which would indicate a majority of the precursor had been consumed. As seen in Table 1, A2–2 resulted in a smaller crystallite size and a lower M_s_ of 60.6 emu/g as compared to A2–24. However, the PDI was significantly reduced under these conditions. Thus, it was thought that this level of monodispersity would provide adequate seeds to use a modified seed growth synthesis to increase the crystallite size. The primary difference between traditional seed growth processes and the modification reported here is in the addition step. Traditional methods involve cooling or aging the nanoparticles, followed by washing in organic solvents and drying to a powder to produce the seeds. [59-61] These seeds are then redispersed in their solvent and more iron precursor is added before the temperature is increased back to the reaction conditions. In this modified seed growth the addition of more Fe precursor is performed at the ‘hot’ reaction temperatures, and thus the nanoparticles stay dispersed and remain at temperatures suitable for nucleation and growth. Using a second addition with a 2hour reaction time, A2–2_B2–2, did produce nanoparticles with an increase in overall size (DLS data Table 1), but this did not increase the crystallite size. As discussed above, the Fe precursor concentration and short reaction time provided lower PDI, while the 24-hour step provided a larger crystallite and increased M_s_. Therefore, a series of modified seed growth syntheses were conducted with various combinations of Fe(acac)_3_ concentration and reaction time at the first and second additions (data in Table 1). Keeping the first addition constant at 2 g for two hours, it was found that a 24-hour step was critical for achieving a larger crystallite size and higher M_s_. The sample from this series with the highest crystallite size of 11.1 nm was A2–2_B6–24. Since this was still under our goal of 15 nm, a seed growth with two 24-hour reaction times was attempted to possibly begin with seeds of a larger crystallite size. The ‘hot’ addition would then allow for continued crystal-lite growth instead of just particle growth. A2–24_B2–24 not only resulted in an increased crystallite size of 12.8 nm, but also a decreased PDI of 0.148.A representative transmission electron microscopy (TEM) image of A2–24_B2–24 nanoparticles is shown in Figure S3. This increase in monodis-persity is speculated to be due to the ‘hot’ addition, providing an initial burst nucleation of small nuclei which are subsequently dissolved and grow on the larger seeds already present in solution, in agreement with ‘Ostwald ripening’.[62,63] Additionally, this mechanism of growth can increase the monodispersity of nanoparticles formed. [64] To corroborate this ‘hot’ addition mechanism, a similar reaction was cooled to 30°C before the second addition of iron precursor, A2–24–B2(30)-24. This resulted in nanoparticles with a smaller crystallite size of 9.6 nm and an increased PDI of 0.252, which suggests that the ‘hot’ addition does indeed facilitate the continued crystallite growth and is an important parameter of this synthesis.

The last parameter investigated was reaction temperature. Using a silicon oil bath for precise temperature control, the reaction temperature was varied for the modified seed growth reactions A2–24(175)_B2–24(175), A2–24(185)_B2–24(185) and A2–24(195)_B2–24(195) (Table 1). A2–24(175)_B2–24(175) resulted in a crystallite size of 11.4 nm with a PDI of 0.404. Raising the temperature to 185°C and 195°C was hypothesized to increase the crystallite size and lower the PDI promoting the Ostwald ripening process and providing better separation of nucleation and growth. Indeed, the crystallite size increased to 13.2 nm for A2–24(185)_B2–24(185), and further increased to 15.2 nm for A2–24(195)_B2–24(195). The 195°C reaction also yielded the highest overall particle size and lowest PDI of 0.219 for the temperature series with two additions. Interestingly, a temperature effect was not seen when running only one 24hour reaction with samples A2–24(175) and A2–24(195), evidenced by no significant change in crystallite size (Table 1). This could indicate that the initial reaction step is governed by LaMer growth, being more dependent on concentrations, with the second addition being dominated by Ostwald ripening and leading to more monodispersed nanoparticles. While future studies are necessary to elucidate this point, the parameters studied here provide a range of iron oxide nanoparticles that were examined for RF heating applications.

### 2.3 Radiofrequency Induction Heating Characterization and Assessment

**Figure 2.**
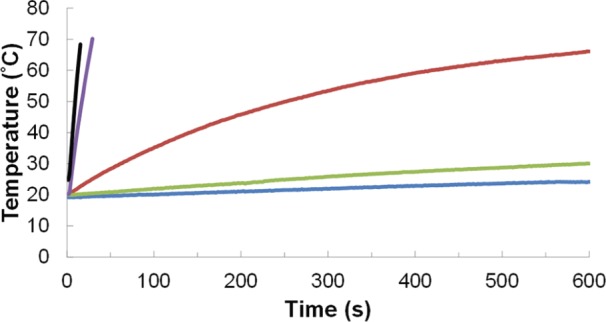
Heating curves of 3 mL of water and iron oxide samples dispersed in 0.25% TMAOH. Concentrations of iron determined by Prussian Blue UV-VIS are 0, 14.48, 15.40, 14.00, and 15.36 mg/mL for deionized water (blue), A2–24 under N2 (green), A2–24 (red), A2–24_B2–24 (purple), and A2–24(195)_B2–24(195) (black) respectively. An alternating magnetic field 175.4 A at frequency of 270 kHz for 600 seconds was used, and the temperature was recorded every 1.4 seconds.

Initial radiofrequency (RF) heating experiments were used to characterize the ability of the iron oxide nanoparticles to heat in solution. For these studies, the nanoparticles were dispersed in a constant volume of 0.25% tetramethylammonium hydroxide (TMAOH) aqueous solution and placed in the AC magnetic field with a magnetic field strength (H) of 37.4 kA/m and a frequency (f) of 270 kHz.RF heating curves of solutions were measured by a fibre optic temperature probe and are shown for deionized water, A2–24 under N2, A2–24, A2–24_B2–24, and A2–24(195)_B2–24(195) in Figure 2.

For comparison between samples, the initial heating rate was calculated from the linear portion of the heating curve. While the mass of iron oxide particles used for each solution was constant, the heating rates were corrected by a Prussian blue assay for the concentration of Fe (Table 1).

**Figure 3.**
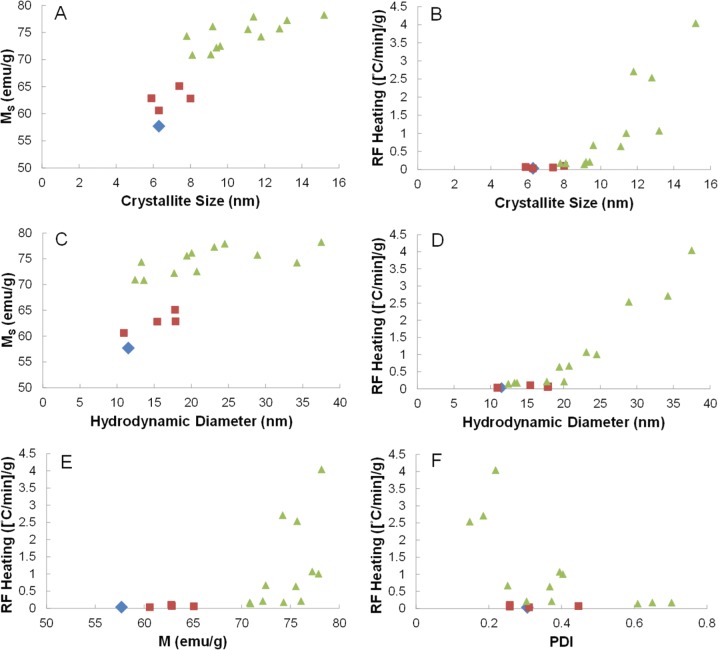
Comparison of syntheses conducted under nitrogen flow (blue), open to air with total reaction times less than six hours (red), and open to air with reactions involving at least one 24-hour reaction step (green). (A, B) Plots of crystallite size versus Ms and RF heating. (C, D) Plots of hydrodynamic diameter versus Ms and RF heating. (E) Plot showing Ms versus RF heating. (F) Plot of polydispersity index versus RF heating.

Compiling all of the data in Table 1, there are several conclusions to be drawn from the relationship between RF heating rate, particle characteristics, and synthetic parameters. First, nanoparticles produced without a 24-hour reaction step all resulted in M_s_ values between 60–65 emu/g as shown in Figure 3A,C,E (red squares). Upon incorporating a 24-hour step, whether at each addition or only the second addition (e.g., A2–2_B2–24), the M_s_ increased to 72–78 emu/g (Figure 3A,C,E – green triangles). While the M_s_ also showed a positive correlation with crystallite size (Figure 3A) and hydrodynamic diameter (Figure 3C), there was no clear correlation between RF heating and M_s_ (Figure 3E). Next, in agreement with the literature, there was a strong correlation between RF heating and crystallite size with a sharp increase as the crystallite size approaches 15 nm (Figure 3B). The data also show a correlation between the RF heating and hydrodynamic diameter (Figure 3D).

While this could be primarily due to the linear correlation between crystallite size and hydrodynamic diameter (Figure S4), it could also suggest that there is an optimal hydrodynamic diameter to provide maximal heat exchange between the particle and the surrounding environment. Further studies will be needed to determine this contribution. Lastly, there is an overall negative correlation between RF heating and PDI, which indicatesthat a system that is more monodispersed has improved heating. However, particle size (both crystallite and hydrodynamic diameter) appears to be the dominant determining factor in optimal RF heating for iron oxide nanoparticles. From the syntheses above, the A2–24_B2–24 synthesis was used for functionalization investigation and *in vitro* studies due to its high RF heating profile (∼2.5[°C/min] /g) and high level of monodispersity (PDI – 0.148), which can enhance overall post-functionalization colloidal stability.

### 2.4 Surface Functionalization and Optimization for Biostability

Modifying the surface of iron oxide nanoparticles is essential for biostability and further conjugation. CMPVA can be attached to the surface of iron oxide through strong interactions with the carboxyl groups.[49] Carboxyl groups not involved in attaching to the surface can be used for further conjugation by reactions such as EDC coupling.

CMPVA was produced through carboxymethylation of polyvinyl alcohol [35] and confirmed by Fourier transform infrared (FTIR) spectroscopy (Figure S5). Previous reported methods for functionalizing the surface ofiron oxide particles with CMPVA required an organic to aqueous phase transfer process using chloroform and 1.5% TMAOH solution.[35] In the studies reported here, the dried particles as prepared in benzyl alcohol were easily dispersed in a TMAOH solution directly and required no opposing phase transfer to remove other capping agents. First attempts for CMPVA functionalization using the previously reported methods proved unsuccessful in obtaining iron oxide nanoparticles with long-term stability in biologically relevant medium (PBS, tissue culture medium, etc.). There could be several factors influencing the particle functionalization efficiency by CMPVA (e.g., particle size, TMAOH concentration, pH, CMPVA concentration, etc.), which the previous work had optimized for their specific material and particle size.[35] Therefore, modifications were made to the surface functionalization process and parameters were investigated to optimize the yield and overall stability of the functionalized iron oxide nanoparticles.

**Figure 4.**
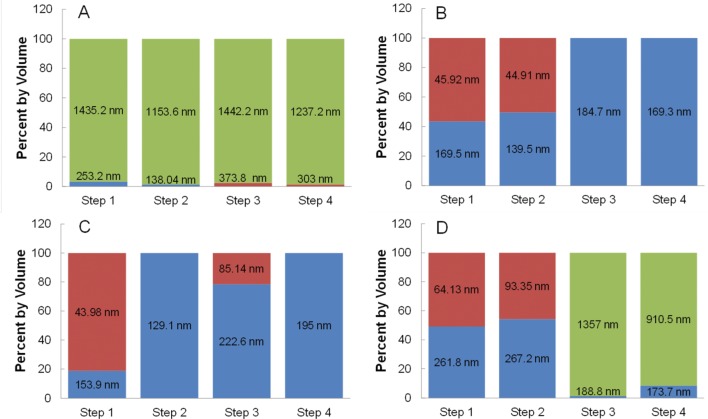
The effect of various concentrations of TMAOH on surface functionalization efficiency of CMPVA. Iron oxide nanoparticles are labelled as either functionalized (blue), inadequately functionalized (red), or aggregated (green) based on their hydrodynamic size (data labels). (A) 0.0625% TMAOH, (B) 0.125% TMAOH, (C) 0.25% TMAOH, and (D) 0.5% TMAOH at several steps in the surface functionalization clean-up process. Step 1 and 2 are before and after the 30k MWCO centrifugal filter. Step 3 and 4 are fractions 1 and 2 of elution from a PD-10 desalting column.

Iron oxide nanoparticles were dispersed at a concentration of 20 mg/mL in 0.0625%, 0.125%, 0.25%, and 0.5% w/w TMAOH and mixed with a constant CMPVA concentration of 20 mg/mL (40:1 CMPVA to iron oxide mass ratio). The initial TMAOH concentration is important in ensuring a stable dispersion of iron oxide nanoparticles prior to mixing with CMPVA. Initial DLS analysis of A2–24_B2–24 dispersed in the various concentrations of TMAOH showed consistent hydrodynamic diameters with a mean of 26.42 ± 1.31 nm (Table S1). In order to analyse the effect of the initial TMAOH percentage on functionalization, DLS was performed at each step of the process and the results are shown in Figure 4. Upon addition of the CMPVA solution, the 0.0625% TMAOH nanoparticle solution became turbid and had a substantial amount of aggregation and precipitation indicated by a PDI value of 0.51 and greater than 95% of the population of particles having an average hydrodynamic diameter over 1000 nm (Figure 4A). This was seen for each step of the functionalization and buffer exchange process. Nanoparticles dispersed in the three higher TMAOH percentages had no aggregation occurring in steps 1 and 2. Since the initial nanoparticle solution was stable, the addition of CMPVA to the 0.0625% TMAOH could lower the pH below what is suitable for colloidal stability of iron oxide nanoparticles. Solutions of CMPVA alone and CMPVA with iron oxide nanoparticles at concentrations identical to reaction conditions were titrated with 6.25% TMAOH (Figure S6). While the pH of the CMPVA alone was at an adequate level for stability throughout the titration curve (8.5 or greater), the addition of iron oxide nanoparticles lowered the pH from 8.68 to 6.89. It was found that a minimum TMAOH concentration of 0.125% was required to reach a pH of 8.5 or greater and keep the nanoparticles dispersed throughout the functionalization with CMPVA. At the highest TMAOH concentration used (0.5%), the iron oxide nanoparticles appeared to have exchanged TMAOH for CMPVA, as seen in Figure 4D steps 1 and 2. However, upon thorough removal of TMAOH and buffer exchange with a PD-10 desalting column (Figure 4D steps 3 and 4) greater than 90% of the population of particles aggregated with an average hydrodynamic diameter over 1000 nm. Furthermore, large amounts of nanoparticles were left on the PD-10 column, indicating a lack of stability as the TMAOH was being removed. This can be explained by inadequate exchange of TMAOH for CMPVA due to a higher concentration gradient keeping the TMAOH on the iron oxide surface. Nanoparticles that remained on the column are thought to have been only stabilized by the presence of TMAOH and thus aggregate and precipitate during the buffer exchange process. Additionally, corroborating evidence for this was seen for nanoparticles in TMAOH solution without CMPVA surface functionalization, which were completely retained on the PD-10 during buffer exchange. Then, as indicated by the DLS data, the populations of nanoparticles with a smaller hydrodynamic diameter are speculated to be functionalized with less CMPVA. These populations of nanoparticles were labelled as ‘inadequately functionalized’ because eluted samples containing this smaller population eventually had some degree of aggregation and a loss of colloidal stability. Therefore, the functionalization was inadequate for long-term stability. It is speculated that the inadequately functionalized nanoparticles aggregate over time due to bridging between nanoparticles at surface regions made available for carboxyl linkage upon removal of the TMAOH. Until the TMAOH was thoroughly removed, this was not apparent, and thus the PD-10 desalting column is an essential clean-up step to improve long-term stability and isolate the stably functionalized population. The two intermediate TMAOH concentrations used both proved successful in functionalizing the iron oxide nanoparticles with CMPVA. The primary difference was seen in step 3, which had a 20% population that was inadequately functionalized for the 0.25% TMAOH (Figure 4C). The re-emergence of inadequately functionalized nanoparticles in this step suggests that the TMAOH concentration was slightly too high for optimal CMPVA exchange. At the 0.125% level, steps 1 and 2 had around a 50:50 population of functionalized and inadequately functionalized nanoparticles (Figure 4B). After steps 3 and 4, the entire population of nanoparticles was functionalized. While both 0.125% and 0.25% TMAOH resulted in functionalized nanoparticles with low PDI values (0.169 and 0.163 respectively) in the final product, the 0.125% had a larger population of functionalized nanoparticles initially (step 1) and thus a higher overall yield. Therefore, 0.125% was determined to be the optimal of the four tested concentrations of TMAOH for surface functionalization with CMPVA.

Next, it was important to test whether or not the above conditions would work with different size nanoparticles. The largest nanoparticles synthesized were A2–24(195)_B2–24(195) with a crystallite size of 15.2 nm and a hydrodynamic diameter of 37.5 nm compared to 12.8 nm and 28.9 nm respectively for the A2–24_B2–24 particles. They were first functionalized under the same optimal conditions determined above (0.125% TMAOH, 20mg/mL CMPVA and the same mass ratio of CMPVA to nanoparticles). This resulted in nanoparticles that were functionalized with CMPVA, but with hydrodynamic diameters larger than desired (average 310.97 ± 51 nm) which resulted in aggregation and precipitation of the final product. Since there was an adequate amount of TMAOH present to keep the nanoparticles stable for CMPVA functionalizing, it was thought that the concentration of CMPVA was too high, causing more crosslinking between particles and leading to larger hydrodynamic diameters. At the same mass, the smaller nanoparticles would have both a higher total surface area and there would be a higher total number of nanoparticles. Therefore, calculations were performed to determine an approximate ratio between the number of CMPVA molecules and both the number of iron oxide nanoparticles and total nanoparticle surface area used in the previous successful functionalization. To simplify calculations of the volume and surface area, the equations for a sphere were used, since TEM analysis revealed a spherical morphology. Calculations were first performed using the diameter determined by the crystallite size (12.8 or 15.2 nm) as measured by XRD and performed using the hydrodynamic diameter (28.9 and 37.5 nm) as measured by DLS. Next, the number of particles in 10 mg was estimated using the calculated volume and density of iron oxide (Fe_3_O_4_ – 5.17 g/cm^3^). The number of particles was then used to calculate the total surface area of 10 mg of nanoparticles. A total of 400 mg of CMPVA was used originally to surface functionalize the 12.8 nm crystallite nanoparticles. Thus, 400 mg was converted to an approximate number of CMPVA polymer molecules using the molecular weight of PVA (6000 g/mol) and Avogadro's number. Then, the number of CMPVA molecules per number of nanoparticles or total surface area in 10 mg was calculated and converted to a required concentration to match the original ratio of CMPVA to nanoparticles or total surface area for the A2–24_B2–24 sample. In this regard, using the increase in crystallite size from 12.8 to 15.2 nm yielded a required concentration of 12.4 mg/mL, and using the increase in hydrodynamic diameter from 28.9 to 37.5 nm yielded a required concentration of 10.7 mg/mL. In relation to surface area, the required concentrations were 17.1 mg/mL and 16.2 mg/mL, respectively. These values were averaged in the following results as the effect of the relative size increases were in close agreement. It was determined that the optimal conditions contained 2.28×10^4^ CMPVA molecules for every one nanoparticle and a ratio of CMPVA molecules to total nanoparticle surface area of 4.43×10^19^. Using these values to adjust for the lower number of nanoparticles and surface area when using the same mass of the larger nanoparticles, A2–24(195)_B2–24(195), two functionalization attempts were conducted at 11.5 mg/mL and 16.7 mg/mL instead of the previous 20 mg/mL. The functionalization reaction matched for the number of nanoparticles (11.6 mg/mL) resulted in functionalized nanoparticles with a PDI of 0.206 and with an average hydrodynamic size of 199.97 ± 34.04nm (50%) and 54.79 ± 24.53 nm (50%) (Figure S7). While these numbers suggest that there is a split in volume percentage of larger clusters and smaller clusters functionalized with CMPVA, they are, as expected, slightly larger than the values in Figure 4, and remained stable in various media. The reaction based on total surface area resulted in the majority of particles having a hydrodynamic diameter approaching 1000 nm, indicating aggregation and cross-linking. It is therefore apparent that when functionalizing different sizes of nanoparticles the concentration of CMPVA should be adjusted to account for the number of CMPVA molecules relative to the number of nanoparticles.

### 2.5 In vitro Studies

With the optimal nanoparticle and surface functionalization methods for stability, some initial pilot experiments were conducted to verify that the nanoparticles could be used to deliver hyperthermia doses to kill glioblastoma tumour cells. For these *in vitro* studies, the functionalized product of A2–24_B2–24 was chosen based on its high RF heating profile (∼2.5[°C/min] /g) and high level of monodispersity. M059K cells were either treated with PBS or iron oxide nanoparticles for 24 hours. Studies have shown that nanoparticles are taken up by cancer cells through various mechanisms, such as endocytosis, electrostatic interactions, or phagocytosis.[65-67] When harvested, the cells were washed thoroughly with PBS and placed in fresh medium to provide one group without any nanoparticles (no particle control group – blue) and one group ‘loaded’ with iron oxide nanoparticles either surface bound or internalized (particle group – red). Based on cellular uptake studies (Figure S8), this group had roughly 0.2 μg of Fe per 100,000 cells. A third group was prepared by taking particle treated cells and adding medium that was loaded with iron oxide nanoparticles (extra particle group – green) at a concentration that would produce measureable heating of the cell suspension. With the additional ∼325 μg of Feloaded in the medium, this group had 99.94% of the iron oxide nanoparticles ‘external’ and 0.06% (0.2 μg/325.2 μg) ‘internal’. Thus, the particle treated group could be considered ‘internal’ heating and the extra particle group will have both ‘internal’ and ‘external’ heating, with the ‘external’ heating being dominant.[65,66]

**Figure 5.**
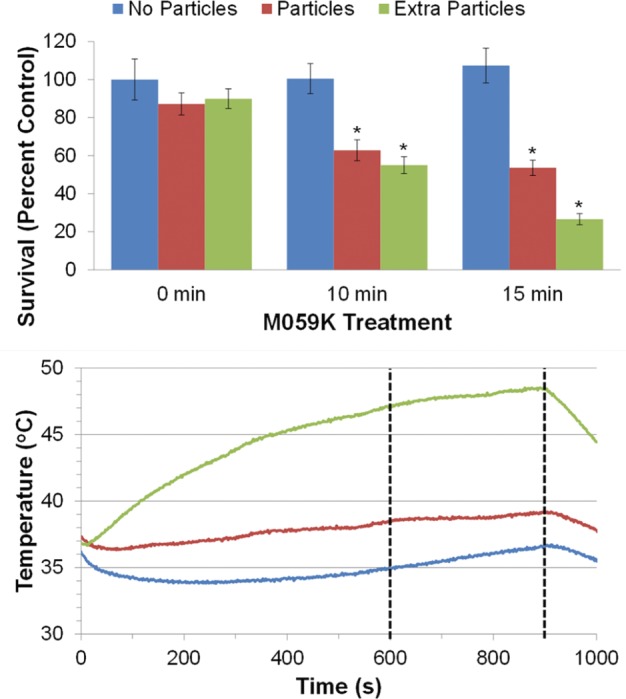
Effect of RF heating (H=42.7 kA/m at f=200.2 A) on M059K cell survival. (Top) The survival of M059K cells treated with either no particles (blue), particles (red), or extra particles (green) and subjected to RF heating for 0, 10, or 15 minutes. The * indicates groups significantly different from the control, as determined by ANOVA and Dunnett two-sided post hoc test at a 0.05 significance level. (Bottom) The respective heating curves for no particles (blue), particles (red), and extra particles (green). Dashed line indicates ten and 15 minutes of heating.

Firstly, as can be seen in Figure 5 (top), the M059K cells that were not exposed to the RF heating showed no significant change in survival between the three treatment groups, indicating that the two iron oxide nanoparticle treatments did not have a significant toxicity alone (see Table S2, S3, and S4 for statistical analysis results). Next, the cells without iron oxide nanoparticles showed no change in survival while being exposed to the RF AC magnetic field for ten and 15 minutes. Furthermore, the temperature profile of the cell suspension during the ten and 15 minute RF treatment in Figure 5 (bottom, blue line) showed little deviation from 37°C. However, RF treatment of both iron oxide nanoparticle treated groups showed a significant decrease in cell survival at ten minutes. While this is the expected result for hyperthermia treatment, the interesting aspect can be seen when comparing the cell suspension temperature profiles (Figure 5 – bottom). The cell suspension of the particle treated group (red line) only reached 38.5°C at ten minutes but showed similar cell killing to the extra particle group (green line) that reached 47.1°C. Then, at 15 minutes, both particle treated groups also showed significantly more cell killing compared to the control group (no particle – 0 min). Although at 15 minutes the extra particle group reached thermal ablation range at 48.4°C compared to just 39.1°C for the particle group and appears to have more cell killing, pairwise comparison of the means (ANOVA – Tukey HSD post hoc test – see Table S5) was not significant. Further experimental replicates (higher N) may reveal a significant difference between these groups, which would be expected due to the greatly different external temperatures reached. These initial results demonstrate that tumour cells can be treated with iron oxide nanoparticles sufficiently to provide hyperthermia effects upon RF heating treatment. They also suggest a potential difference between the ‘internal’ and ‘external’ heating modes, which needs to be investigated further to elucidate the benefits and mechanism of cell death involved.

## 3. Conclusion

Iron oxide nanoparticles were successfully produced at a variety of sizes by use of a modified seed growth approach requiring just the iron precursor and solvent, and only one subsequent hot addition of precursor without intermediate wash steps. With the parameters investigated, it appears that combining factors that influence the LaMer model for growth with other factors that drive the Ostwald ripening model may lead to further size control and monodispersity with the benzyl alcohol synthesis system. While the RF heating investigation revealed increased heating, as expected while approaching the 15 nm crystallite, further studies will strive to increase the crystallinity of iron oxide nanoparticles without large changes in the overall particle size. This will provide a more comprehensive answer to the optimal combination of crystallite and particle size for RF induction heating applications. The surface functionalization optimization yielded both a better understanding of the impact of the TMAOH concentration, and the ability to tune the CMPVA concentration for stabilizing different sizes of iron oxide nanoparticles for biological applications. The CMPVA functionalized iron oxide nanoparticles produced under these conditions were stable for longer than six months. Future studies of CMPVA surface functionalization will aim to further optimize the amount of time required for adequate surface functionalization and probe for possible effects on nanoparticle stability due to shortening or prolonging the surface functionalization step. Then, the *in vitro* studies, in total, suggest two possibilities that need to be investigated further. First, the comparable cell killing at largely different temperatures could indicate a difference in the mechanism of cell death for internal versus external heating modes. This could lead to other therapies having a synergistic interaction with the internal nanoparticle delivered hyperthermia that are not currently known to interact with traditional hyperthermia. Furthermore, the ability to kill tumour cells without a significant increase in the temperature of the surrounding environment or tissue could make the nanoparticle-delivered hyperthermia more viable for treating difficult cancers like glioblastoma, while limiting the risk of normal tissue damage. These two points will need to be investigated fully to further aid translation into the clinical setting.

## 4. Experimental Section

### 4.1 Synthesis of Iron Oxide Nanoparticles

All chemicals and materials were used as received. Iron oxide nanoparticles were synthesized under nitrogen flow or open to air in a two-neck 100 mL round bottom flask (Chemglass) equipped with a coil style reflux condenser (Chemglass). First, iron (III) acetylacetonate (Fe(acac)_3_) (2, 4, or 6 g) (Acros Organics, 99+%) was dissolved in benzyl alcohol (20 mL) (Alfa Aesar, 99%) under constant magnetic stirring. The solution was stirred vigorously and immediately heated to reflux. Upon colour change from dark red to black, the reactions were carried out for two or 24 hours. The reaction was removed from heat and stirred for 15 minutes to allow for cooling. The resultant iron oxide nanoparticles were precipitated in acetone (Fisher Scientific, ACS grade) and extracted by magnetic separation. Washing with acetone was repeated 3–5 times with brief sonication (Cole Parmer, Ultrasonic Cleaner 8892) between washes. Flowing nitrogen was used to dry the nanoparticle product to a fine powder.

For the modified seed growth procedures, Fe(acac)_3_ (2 g) was dissolved in benzyl alcohol (20 mL) in a round bottom flask under a coil style reflux condenser, stirred vigorously, and heated to reflux, as described above. At two or 24 hours of reaction time a second addition of Fe(acac)_3_ (2, 4, or 6 g) was added, as a solid powder, directly to the hot reaction and continued to react for two or 24 hours. For reactions where precise control of temperature was required, the heating mantle (Thermoscientific, electrothermal heating mantle) was replaced with a silicon oil bath (Alfa Aesar) and temperature controlled by the magnetic stirring hot plate (VWR, VMS-C7) equipped with a temperature control unit (VWR, VT-5 S40). To determine important temperature thresholds, the temperature and colour of the solution was monitored and recorded every minute until reaching the desired reaction temperature and the solution colour changed completely to black, indicating high levels of nanoparticle formation. Reactions were heated at the highest ramp rate obtainable by the heating mantle or hot plate and oil bath. Products from the seed growth were cooled and washed under the same conditions as all other reactions listed above.

### 4.2 Synthesis of Carboxymethylated Polyvinyl Alcohol (CMPVA)

CMPVA was synthesized by reacting polyvinyl alcohol (PVA) (Polysciences Inc., MW 6000, 80 mol% hydrolysed) with bromoacetic acid (Alfa Aesar, 98+%) in the presence of base as previously described.[35] First, three solutions were made as follows: Solution A – PVA (5 g) dissolved in H2O (50 mL), Solution B – sodium hydroxide (NaOH) (5.324 g) (Alfa Aesar, pearl, 97%) dissolved in H_2_O (25 mL), and Solution C bromoacetic acid (11.575 g) dissolved in 70% ethanol (EtOH) (200 mL) (Sigma Aldrich, 200 proof for molecular biology). Next, NaOH (3.33 g) was slowly dissolved in Solution C. Solution B was added to Solution A slowly under constant stirring at 50°C. This was followed by drop wise addition of Solution C and reacted under a reflux condenser at 50°C for five hours to carboxymethylate the PVA. The pH of the synthesized CMPVA was adjusted to ∼6.0 with hydrochloric acid (HCl) (1 M) (Fisher Scientific, Optima) and precipitated with cold EtOH (Pharmco-AAPER, 190 proof ACS/USP grade). CMPVA product was centrifuged at 3000xg for 15 minutes (Thermo Scientific, Sorvall Legend ×1R centrifuge) and washed 3–6 times with EtOH. The CMPVA was then dried in a vacuum oven at 50°C for one week.

### 4.3 Surface Functionalization of Iron Oxide Nanoparticles with CMPVA

First, dry iron oxide nanoparticles (40 mg) were dispersed in aqueous solutions of tetramethylammonium hydroxide (TMAOH) (Alfa Aesar, 25% w/w aqueous electronic grade 99.9999%) (2 mL; 0.0625%, 0.125%, 0.25%, or 0.5% w/w). The solution was not magnetically stirred at any point in the functionalization process, in order to limit any aggregation from magnetic fields that might occur. The nanoparticle solution was sonicated for 180 minutes and allowed to sit overnight. Then, the nanoparticle solution (0.5 mL) was added to a mixture of CMPVA (10 mL, 40 mg/mL) and H_2_O (9.5 mL). Subsequently the solution was sonicated for 180 minutes and allowed to sit for one week. One week was chosen for this work as initial functionalization studies were carried out and observed for two weeks. During this two-week time frame, the particles remained slightly turbid after 24–48 hours, but were visibly clear after one week of reaction. The solution was then centrifuged in a 30K molecular weight cut-off centrifugal filter (PALL, Macrosep® Advance Device) at 4000xg for one hour to concentrate the nanoparticles and remove excess CMPVA. The concentrated nanoparticle solution was transferred to a new vial and sonicated for 15 minutes to ensure complete redispersion. Next, the CMPVA functionalized iron oxide nanoparticle solution was loaded on to a PD-10 desalting column (GE Healthcare, Sephadex® G-25 medium), equilibrated with H2O (Fisher Scientific, Optima ® LC/MS grade) to remove any remaining TMAOH and non-functionalized or aggregated nanoparticles. This was followed by an additional concentration step using the same 30K centrifugal filter as above spinning at 4000xg for one hour and sterile filtering using a 0.2 μm sterile filter (PALL, Acrodisc® Supor® membrane) in a biosafety laminar flow hood. The resulting sterile nanoparticles were stable in water and PBS.

### 4.4 Characterization

#### 4.4.1 X-ray Diffraction

Powder x-ray diffraction (XRD) patterns were obtained on a PANalyticalX'Pert Pro Materials Research Diffractometer. Dried samples (∼300 mg) were mildly ground to obtain a fine powder. The fine powder was transferred to a low background silicon disk. XRD patterns were scanned at 20–80° 2θ using a Cu Kα x-ray source and evaluated using X'Pert High Score Plus software. The Scherer equation was used to calculate the crystallite size from peak broadening of diffraction peaks.

#### 4.4.2 Dynamic Light Scattering

The hydrodynamic diameters and polydispersity indexes of iron oxide nanoparticles were analysed at ambient conditions using a Malvern ZetasizerNano-ZS (Malvern Instruments, U.K.). The DLS light source used was a He-Ne laser (633 nm, max 4 mW). Iron oxide nanoparticles (20 mg/mL) were dispersed in TMAOH solution (0.25%) and sonicated for 180 minutes. After sitting overnight a 1:100 dilution (1 mL) was made for DLS analysis. Samples were then transferred to a low volume disposable cuvette and hydrodynamic diameter and PDI values were calculated as an average of five runs containing 11 measurements per run.

#### 4.4.3 Vibrating Scanning Magnetometry

Magnetic characteristics were probed using a VersaLab 3 Tesla Cryogen-Free Vibrating Sample Magnetometer (VSM) (Quantum Design). Samples were prepared by weighing dry samples (5–15 mg) and sealing in a sample capsule (Quantum Design). VSM sample capsules were loaded and scanned for offset at 35 mm. Moment versus field measurements were conducted at <50 Torr purged pressure, a sweep rate of 150 Oersted/second (Oe/s) with no automatic centring and scanning five quadrants from 0 Oe to 15,000 Oe (H_max_) to −15,000 Oe (H_min_). Saturation magnetization was determined from the magnetization versus magnetic field strength plots at H_max_ or H_min_. Samples were mass corrected with thermogravimetric analysis (TGA).

#### 4.4.4 Thermogravimetric Analysis

To determine mass corrected values, thermogravimetric analysis (TGA) was run on a Q5000 TGA (TA Instruments). Dry samples (5–50 mg) were loaded onto platinum pans and the temperature was ramped at 10°C/min from room temperature to 150°C and held isothermal for 15 minutes. Subsequently, ramping was continued at 10°C/min to 400°C and held isothermal for 60 minutes. TGA was run under a nitrogen flow rate of 25 mL/min.

#### 4.4.5 Transmission Electron Microscopy

Brightfield transmission electron microscopy (TEM) images of iron oxide nanoparticles were obtained with a Zeiss LIBRA® 120 PLUS TEM. Samples were prepared for TEM by drying 1:10 dilution of iron oxide nanoparticles in 0.25% TMAOH solution (2 mL; 20 mg/mL) on copper TEM grids (Ted Pella Inc., 200 mesh Formvar carbon type B). Images of CMPVA functionalized iron oxide nanoparticles were loaded at a 1:10 dilution after all clean up and filtering processes described above. Nanoparticle size measurements were performed using Image J software.

#### 4.4.6 Fourier Transform Infrared Spectroscopy

Successful synthesis of CMPVA was determined by Fourier transform infrared (FTIR) spectroscopy using Thermo Scientific Nicolet 6700 equipped with a smart iTR for attenuated total reflectance (ATR) of samples. Dry CMPVA and PVA samples were pressed onto the diamond crystal and analysed using single bounce ATR.

### 4.5 Radio Frequency Heating Experiment

The heating properties of iron oxide nanoparticles synthesized by different parameters were investigated using 1.2–2.4 kW EasyHeat induction heating system with a coil designed at a setpoint of 200 A to run at 1222 W and frequency (f) of 269 kHz to produce an alternating magnetic field with a magnetic field strength (H) of 37.4 kA/m at 175.4 A. The temperature of the solution being exposed to the RF AC magnetic field was measured in situ with an OpSens-fibre optic temperature sensor and recorded by SoftSens software. Initial tests were performed on iron oxide nanoparticles in 0.25% TMAOH aqueous solution (3 mL; 20 mg/mL). The RF heating was conducted at 175.4 A, and H=37.4 kA/m for 600 seconds and the temperature was recorded every 1.4 seconds. To account for convection heating, water (3 mL) was measured under the same conditions. The temperature rise was constant over the entire 600 seconds with a dT/dt value of 0.549°C. This value was used to correct the initial linear temperature rise of RF heating of iron oxide TMAOH samples. RF heating values are corrected for the concentration of iron as determined by Prussian Blue assay. A standard curve was produced by Prussian Blue UV-Vis absorption assay (λ=715 nm) with a Fe ICP standard (Alfa Aesar, Iron, plasma standard solution, Specpure®, Fe 1000 μg/mL) and UV-Vis absorption with a Nanodrop 2000c spectrometer (Thermo Scientific). The RF heating samples were first diluted 1:100. Then, the samples (10 μL) were mixed with HCl (10 μL; 2%) (Electron Microscopy Sciences) and Prussian Blue (20 μL; 2%) (Electron Microscopy Sciences). After exactly 15 minutes of incubation at room temperature UV-Vis absorption of prepared samples (2 μL) was measured with no baseline correction. This same method was used to assess the cellular uptake for the CMPVA functionalized iron oxide nanoparticles used in the cell survival studies. Medium samples were prepared for concentration assessment similar to the above solutions. Cell pellets were collected and subjected to lysis buffer (Sigma Aldrich, CelLytic M) prior to preparation for Prussian Blue addition, in order to release the nanoparticles into solution. Further details are provided in Supporting Information.

The solution of CMPVA functionalized iron oxide nanoparticles (650 μL) was tested to determine optimum heating power and time for cell survival experiments. The solution of iron oxide nanoparticles at 37°C were heated at 200.2 A and 230.4 A for 1200 seconds to determine which temperatures were reached over time. Heating at 200.2 A (H=42.7kA/m, f=270 kHz) for 1200 seconds resulted in reaching temperatures in the moderate hyperthermia range. To ensure that heating occurs mainly due to coupling to the alternating magnetic field and not convective heat flow from the coil cell media, 650 μL was heated in a 1.5 mL microcentrifuge tube and the temperature increase was monitored as a control.

### 4.6 Cell Survival Study

M059K cells (American Type Culture Collection, glioblastoma) were cultured in 1:1 Modified DMEM/F-12 medium (Hyclone, 0.1 μm sterile filtered) containing 10% foetal bovine serum (FBS) (Seradigm, ultimate grade, triple 0.1 μm sterile filtered), 1% antibiotic/antimycotic solution (Hyclone, 10,000 U/mL Penicillin G, 10,000 μg/mL Streptomycin, 25 μg/mL Amphotericin B, 0.2 μm filtered) in an incubator at 37°C and 5% CO_2_. Cells were grown to ∼80% confluence in T75 flask (Greiner Bio-One, CELLSTAR®, red filter cap, sterile) and passaged by washing with Dulbecco's phosphate buffered saline (DPBS) (Corning, cellgro, without calcium and magnesium, sterile) and trypsinised with 0.25% trypsin solution (Hyclone). For the RF heating experiment, cells were harvested and counted using a handheld automated cell counter (Millipore, Scepter™), followed by plating 800,000 cells per flask in two T25 flasks (Greiner Bio-One, CELLSTAR®, red filter cap, sterile) in culture medium (5 mL). Once the cells had attached (overnight, ∼16 hours) one flask was treated with DPBS (250 μL) while the other was treated with CMPVA functionalized iron oxide nanoparticles (250 μL; 2 mg/mL of Fe) and placed back in the incubator for 24 hours. Cell medium was removed and cells were washed with DPBS three times to remove excess iron oxide nanoparticles. The cells were trypsinised and counted to obtain the cell count per mL at the start of the heating experiment. Then, aliquots of the cell suspension (500 μL; ∼100,000 cells) from the DPBS treated group were transferred into three microcentrifuge tubes and aliquots of the nanoparticle treated group (500 μL) were transferred into six microcentrifuge tubes. Next, extra nanoparticles (150 μL; 2 mg/mL of Fe) were added to three of the six microcentrifuge tubes of the particle treated group. One tube from each of the three groups was subjected to either no heating, heating for 600 seconds, or 900 seconds at 200.2 A, 1284 W with a 269 kHz coil (H = 42.7 kA/m). After heating, the cells were diluted to 150 cells per mL to plate 300 cells in medium (2 mL) per well of a six-well plate (Greiner Bio-One, CELLSTAR®) for a colony formation assay. These plates were incubated at 37°C for two weeks and colonies were then counted by staining with crystal violet.

### 4.7 Statistical Analysis

When applicable, values are presented as mean ± standard error. Treatment groups in the cell survival experiment were compared to the control using ANOVA and Dunnett two-sided post hoc test at a 0.05 significance level. For comparison between individual treatment groups, a Tukey HSD post hoc test at a 0.05 significance level was utilized. Analysis was performed using IBM SPSS Statistics 22 software.

## 5. Supporting Information

Supporting information is available online or from the author.
